# Quantitative Approach to Fish Cytogenetics in the Context of Vertebrate Genome Evolution

**DOI:** 10.3390/genes12020312

**Published:** 2021-02-22

**Authors:** Veronika Borůvková, W. Mike Howell, Dominik Matoulek, Radka Symonová

**Affiliations:** 1 Faculty of Science, University of Hradec Kralove, 500 03 Hradec Kralove, Czech Republic; veronika.boruvkova@uhk.cz (V.B.); dominik.matoulek@uhk.cz (D.M.); 2 Department of Biological and Environmental Sciences, Samford University, Birmingham, AL 35226, USA; wmhowell@samford.edu; 3 Department of Bioinformatics, Wissenschaftszentrum Weihenstephan, Technische Universität München, 85354 Freising, Germany

**Keywords:** GC content, chromosome size, linkage group, microchromosomes, GC-biased gene conversion

## Abstract

Our novel Python-based tool EVANGELIST allows the visualization of GC and repeats percentages along chromosomes in sequenced genomes and has enabled us to perform quantitative large-scale analyses on the chromosome level in fish and other vertebrates. This is a different approach from the prevailing analyses, i.e., analyses of GC% in the coding sequences that make up not more than 2% in human. We identified GC content (GC%) elevations in microchromosomes in ancient fish lineages similar to avian microchromosomes and a large variability in the relationship between the chromosome size and their GC% across fish lineages. This raises the question as to what extent does the chromosome size drive GC% as posited by the currently accepted explanation based on the recombination rate. We ascribe the differences found across fishes to varying GC% of repetitive sequences. Generally, our results suggest that the GC% of repeats and proportion of repeats are independent of the chromosome size. This leaves an open space for another mechanism driving the GC evolution in vertebrates.

## 1. Introduction

The quantitative approach in fish cytogenetics, or more precisely in cytogenomics, has so far been rather neglected and the same can be said for vertebrates in general. This is due to the lack of available data. The traditional cytogenetics prevailing during the last decades has mostly used a qualitative approach, which was indispensable for the exploration of chromosomal traits generally. With the still accelerating progress of genome sequencing, a large body of evidence on base composition, i.e., the proportion of guanines and cytosines in DNA (GC%), became available also in non-model vertebrates. In parallel, the Animal Genome Size Database [1] is another valuable resource of information useful particularly to estimate the completeness of genome assemblies. Only this recent development enabled us to tackle compositional cytogenomics of vertebrates from a quantitative viewpoint.

There were attempts to quantify results of the mostly qualitative (molecular) cytogenetics during all the phases of its development. With the first human karyotypes being presented during the early 1960s, it was not surprising to see an explosion of karyotypes being rapidly published in other living groups of organisms, especially vertebrates. However, this was before chromosome banding techniques (G-, Q-, C-, R-, AgNOR, etc.), karyotypes were simply grouped according to their size and the position of the centromere. Quantitatively, one could still determine a number of chromosomal features: 1. The chromosome number of a species; 2. the position of the centromere; 3. often the position and number of the secondary constriction(s) = nucleolus organizer regions; 4. a measurement of the chromosome long arm length; 5. measurement of the short arm length; 6. measurement of the total chromosome length (TCL); 7. the long arm/short arm ratio (this was important as it pinpointed the position of the centromere along the chromosome) [2]; 8. the total haploid chromosome length (THCL); 9. the fundamental number (FN) of a karyotype (determined by counting the chromosome arms). Therefore, the FN ≤ 2 × 2 n, with the difference depending on the number of mono-armed chromosomes present (telocentric or acrocentric); 10. measurement of the centromeric index (CI), which is the length of the short arm divided by the total length of the chromosome × 100. The CI proved to be a very valuable quantitative measure for describing the shape of a chromosome, especially before chromosome banding methods [2,3].

Since the chromosomes of the vast majority of fish species could not be banded, especially G-banded, most of the authors of early fish karyotype papers included from one to several of the above counts and measurements in efforts to quantify their findings. The difficulty in obtaining chromosome banding patterns in fishes is likely due to their small size relative to that of higher vertebrates, and that, unlike birds and mammals, whose genomes can be divided into multiple GC-rich and AT-rich segments, fishes have been suggested to have little compartmentalization of their genomes by base composition [4,5,6,7], etc. Other quantitative traits were developed and have often been investigated in fish separately: Chromosome numbers, particularly in a phylogenetic context [8]; genome size expressed as the C-value based on the Animal Genome Size Database [1], as related to the population size [9], and to the nucleotypic effect [10,11].

The base composition at the level of fish chromosomes has so far been addressed in two model species in relation to isochores [12]. In two more fish species, the base composition was analysed in the entire genome [13,14]. However, these quantitative cytogenomic traits, particularly at the chromosome level, are crucial inputs in the analyses of mechanisms driving the regional GC% and the AT/GC heterogeneity in mammals and birds vs. the AT/GC homogeneity in lower vertebrates. Namely, one of the currently most accepted concepts trying to elucidate the aforementioned questions is the GC-biased gene conversion, gBGC [15,16,17]. This concept explains the increased GC% and its heterogeneous organisation in mammals and birds by the recombination rate (RR) per megabase pair (Mb; recently reviewed by [18]). Here, the chromosome or chromosome arm size plays an important role as there is at least one crossing-over (i.e., one recombination event) per one chromosome arm in bi-armed chromosomes and per chromosome in mono-armed chromosomes [18,19]. Among vertebrates, the efficiency of gBGC on the GC% has been investigated in great detail mostly in the coding (i.e., exonic) regions and above all in mammals [20,21,22] and birds [23,24], but also in reptiles [25,26]. The same applies for the online available GCevobase, an evolution-based database for the GC content in eukaryotic genomes displaying GC contents for all the annotated coding sequences from Ensembl [27]. However, the coding sequences make up not more than 2% of the human genome and similarly small fractions in other vertebrates (Matoulek et Symonová, unpublished data). Therefore, these highly detailed and sophisticated analyses referenced above could not catch the compositional evolution along large genome fractions as, e.g., the far more abundant repetitive fraction. Another approach represented by Frenkel et al., employed a far higher resolution beyond the chromosome, thus omitting their importance. They also investigated a limited number of species available at that time [28]. A more relevant study by Li and Du already focused on the chromosome level, however, dividing animals into mammals and non-mammals blurred the lineage specific traits together with the low number of analysed species available at that time [29]. Later, fully irrespective of GC% and its organisation, fishes were demonstrated to have the highest recombination rate among vertebrates [19] even without including the information on chromosome arms (FN) numbers, which would further increase the acquired values. This shows an urgent need to revisit this issue from another, so far unexplored viewpoint.

GC% is linked to the genome size [30] and hence, chromosome counts are related to the genome size and chromosome size as well as their morphology (bi-armed vs. mono-armed; [19]). The genome size shows a clear positive association with the proportion of repetitive elements, particularly of transposable elements (TEs [31]; etc.).

In terms of genome composition, we need to distinguish the overall genomic GC-richness or GC content, i.e., the percentage of G + C (GC%) from the avian or mammalian situation recognized as the AT/GC heterogeneity [32], known also in non-teleost gars [33] and further confirmed by [34] in this special issue. The latter situation is characterized by an alternation of GC-rich and GC-poor regions along chromosomes, thus forming banding patterns upon an AT- and GC-specific staining (for more details, see [34] in this special issue). This AT/GC heterogeneity had been ascribed to a higher diversity of the isochores and their families distinguished according to their GC% [35]. In the AT/GC heterogeneous (mammalian, avian, and gar) genomes, the overall GC% can, however, be even lower than it is in the AT/GC homogeneous (fish) genomes. Considering that all of the currently available vertebrate genome assemblies contain gaps due to either repeat-rich or GC-rich regions [36], fish with GC-rich genomes might actually be even more GC-rich than estimated, and potentially even more GC-rich than mammalian and avian genomes. However, this bias should be comparable for all the genomes along the vertebrate phylogenetic tree. The only difference can be expected in newer improved versions of genome assemblies employing the hybrid approach of sequencing as, e.g., [37] recently in bats.

Our goal in this study is to utilize the increasingly available vertebrate genomes assembled to the chromosome level to assess the role of chromosome size in the overall GC content and in the AT/GC genomic heterogeneity across vertebrates. To do so, we employed our novel Python-based tool EVANGELIST published in this special issue [34]. This tool uses the sliding window approach to visualize and quantify the percentage of repeats (rep%) and GC percentage (GC%) in both repeats and non-repetitive DNA simultaneously along the chromosomes [34]. Employing this tool revealed several interesting quantitative traits at the chromosomal level across the vertebrates. These inputs together with a large-scale meta-analysis of genomic data serve as a test of the concept of GC-biased gene conversion in fishes and in other vertebrates.

## 2. Materials and Methods

We meta-analysed NCBI/genome records (accessed in January 2021) on the genome size, chromosome size, and GC percentage (GC%) of entire genomes and their chromosomes (in the genome assemblies called linkage groups, LGs).

### 2.1. Data Acquisition, Filtering, and Manual Curation

**Figure 1:**
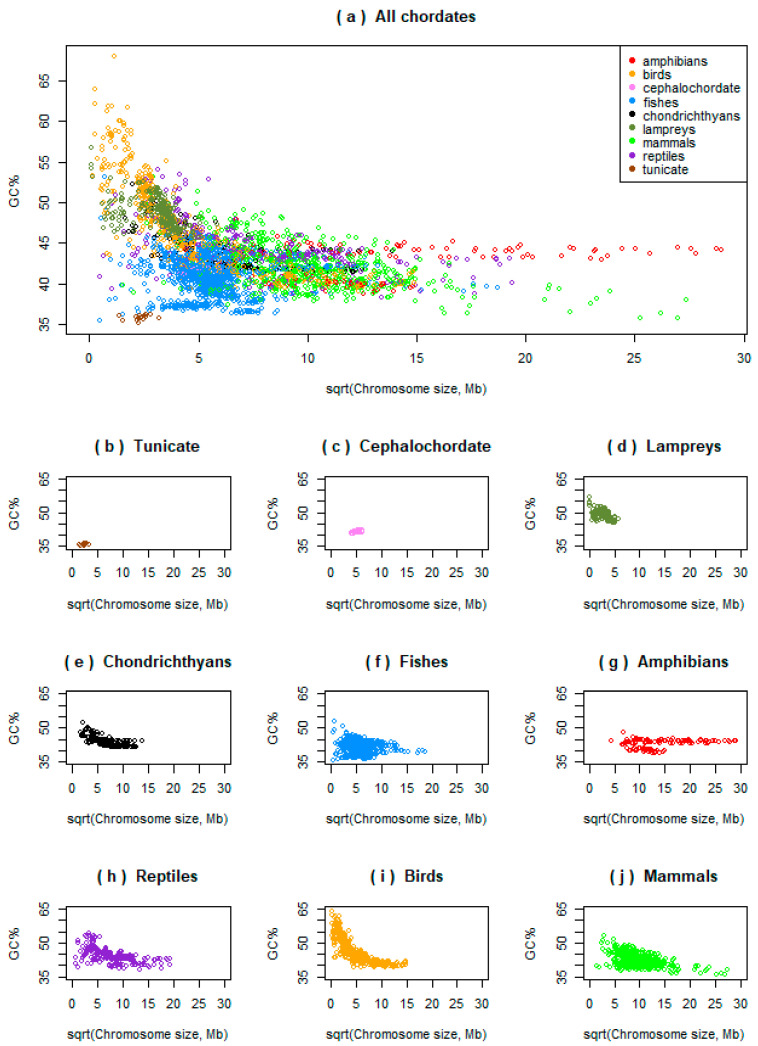
Large-scale overview of the relationships between chromosome size (*x*-axis, square root transformed for a better visualization) and chromosome GC% (*y*-axis) across selected chordates on equidistant axes *x* and *y*. (**a**) All chordates together; (**b**–**j**) major chordate lineages separately ordered phylogenetically. The graph (f), fishes, includes two basal ray-finned species (sturgeon and gar) and teleosts. In (g), the largest genome of axolotl is not visualized, see Figure 4 for amphibians. Each point represents a single chromosome of a species within the given group.

**Figure 2:**
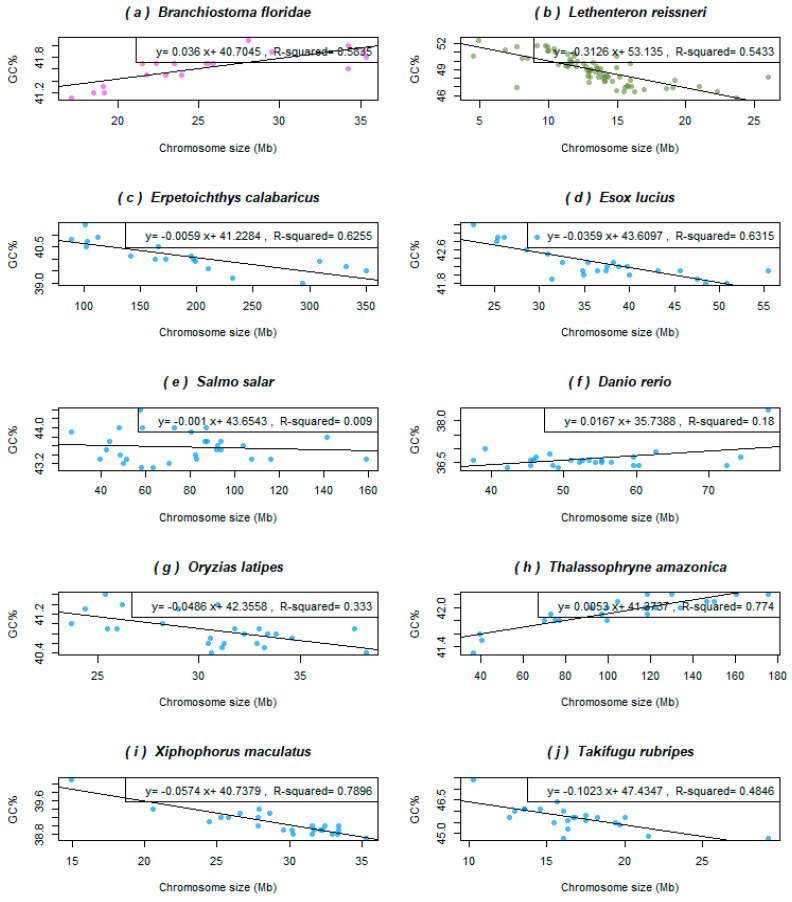
Relationships between GC% and the chromosome size in Mb in selected fish and fish-like species representing different patterns of this association. (**a**) Lancelet as the basal chordate considered the ancestral condition shows a significantly positive association between the chromosome size and GC%; (**b**) lamprey, the extremely GC-rich jawless fish with numerous mostly tiny chromosomes, and reedfish (**c**) with its giant non-teleost genome and extremely large chromosomes both show a significantly negative association; (**d**) the Northern Pike, an important pre-duplication model species for salmonids, shows a significantly negative association, as well; (**e**) in the Atlantic salmon, there is no association between these two measures; (**f**) zebrafish, although the mostly investigated fish model with a larger teleost genome, shows a rather weak positive association; (**g**) medaka, another important model species, shows a weaker negative association; (**h**) toadfish with a surprisingly large teleost genome shows a significantly positive association and the largest teleost chromosome, although no whole genome duplication is known here as, e.g., in salmonids with comparable genome and chromosome size but higher chromosome numbers; (**i**) the Southern platyfish shows the most significant negative association and the typical teleost chromosome sizes; (**j**) fugu represents the compact teleost and vertebrate genomes shows a slightly less prominent but still significantly negative association. The colour code corresponds to Figure 1.

**Figure 3:**
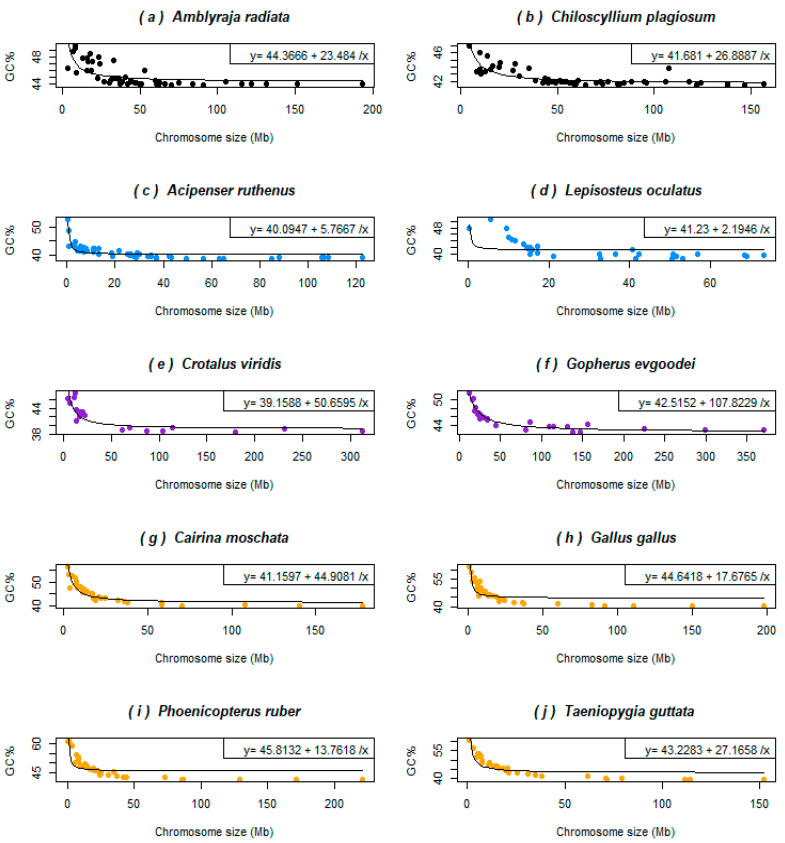
Relationships between GC% and the chromosome size (Mb) in basal fish lineages that all possess microchromosomes show an inverse relationship as do birds and reptiles with microchromosomes. (**a**) The thorny skate, representing the superorder Batoidea, is the GC-richest species in this group; (**b**) the whitespotted bamboo shark represents the superorder Selachimorpha; (**c**) sterlet, one of the only two sequenced sturgeons, with the GC-poorest genome; (**d**) the spotted gar belongs to the infraclass Holostei and is known for its mammalian-like AT/GC compartmentalization; (**e**) the rattlesnake represents a smaller and GC-poorer reptilian genome; (**f**) gopher tortoise represents a larger and more GC-rich reptilian genome; (**g**) the Muscovy duck is the first avian genome compared here; (**h**) chicken represents one of the best assembled avian genomes; (**i**) the American flamingo represents large wading birds; finally, (**j**) the zebra finch with its fifteen genome assemblies available represents songbirds. Violin plots of these species are available in the Supplementary Figure S3. The colour code corresponds to Figure 1.

**Figure 4:**
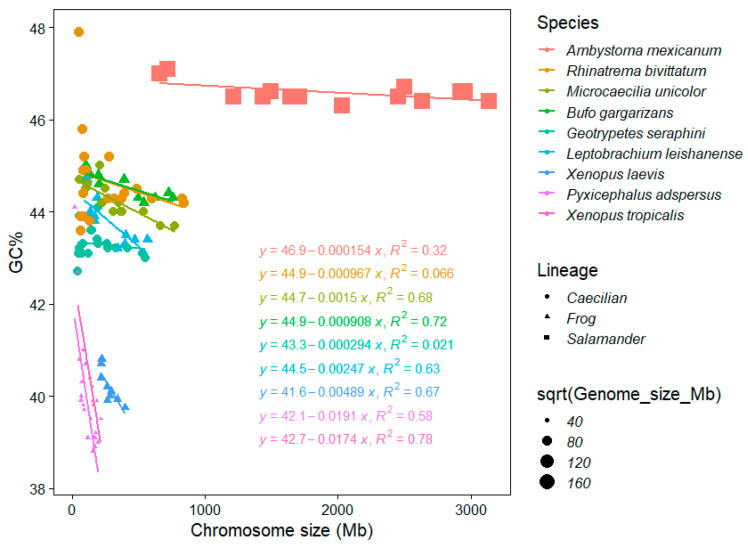
The chromosome size and GC% of chromosomes in currently available amphibians. Nine species ordered according to the size of their entire genomes in the legend and equations, which is also reflected in the point size. Point shapes reflect the main amphibian lineages. Patterns of the associations between the chromosome size and GC% forms three distinct groups partly corresponding to the main lineages and fully corresponding to genome sizes.

At the NCBI webpage (NCBI, 2016) using the available genome selection filters, we selected chordates assembled to the chromosome level. We manually checked the obtained datasets for multiple records (particularly human, dog, primates, and other model species), incomplete assemblies and hybrids. The online filtering yielded 157 fishes, 10 amphibians, 13 reptiles, 66 birds, and 327 mammals. However, upon the manual inspections, the numbers of species usable in our study decreased to 85 mammals, 55 birds, 12 reptiles, nine amphibians, and 118 fishes. Among fishes, there were four chondrichthyans, three lampreys, one lungfish with an incomplete dataset, one sturgeon, one gar, one bichir, and 107 teleosts (lists of species are in the Supplementary). These data were used for Figure 1, Figure 2, Figure 3 and Figure 4 and Figures S2 and S3. These figures were prepared using R [38] with the library scales included in the basic R and further with packages ggplot2, forcats, and ggpmisc, all belonging to tidyverse [39]. There was a bias in mammalian lineages represented among the species assembled to the chromosome level. Namely, some lineages were not represented at all (Afrotheria, Cingulata, Scandentia, Dermoptera, Eulipotyphla, and Pholidota), some were underrepresented (Chiroptera, the second largest mammalian order with only three species available), and some were overrepresented (primates, rodents, and artiodactyls). More details are available in the Supplementary Methods. Therefore, we have selected a subset of 26 mammals trying to compensate for this bias and to avoid the overrepresented species skewing the results. Similarly, in birds, the most abundant order available were Passeriformes and to a less extent also Anseriformes. Hence, we again selected a subset of 18 avian species to avoid any bias towards these orders. However, some avian species, despite being filtered, turned out not to have the required chromosome level assembly available. Finally, we performed a comparable species selection in fish and produced a subset of 49 species representing the orders, as much as possible, and not overrepresenting the better explored ones.

### 2.2. Repeats Analyses and Genome Size Data

**Figure 5:**
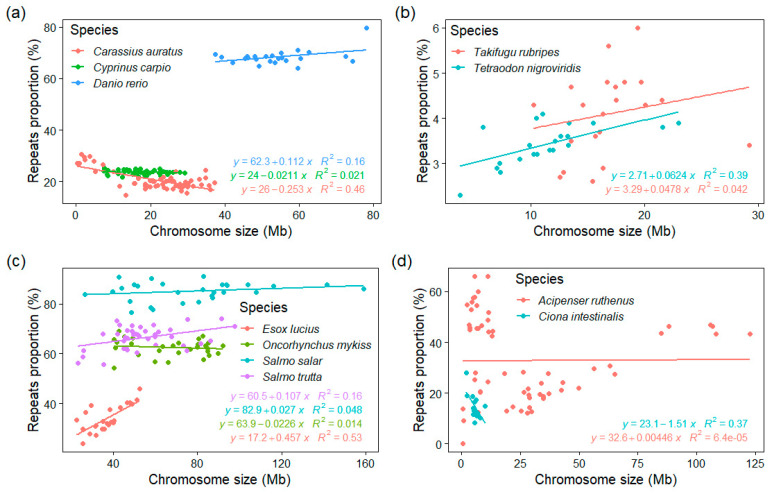
Relationships between the repeats proportion and chromosome size in selected fish species with relevant traits—genome size, chromosome numbers and sizes, and whole-genome duplication or genome compaction underwent. (**a**) In three cyprinids with highly different cytogenomic traits; (**b**) two of the smallest teleost (and vertebrate) genomes show different associations; (**c**) the Northern pike as the pre-duplication ancestor of salmonids shows a clear positive association, while three salmonids show no association with a substantial repeats enrichment; (**d**) the tunicate sea squirt has a chromosome size comparable with microchromosomes of sturgeon, however, differs greatly in proportions of repeats showing a negative association.

To employ the Python-based tool EVANGELIST [34], we have used repeat-masked data from Ensembl to analyse a potential relationship between the GC% and repeats proportion (rep%). This approach was utilized also to address the issue of a potential effect of the decreasing chromosome size (particularly in microchromosomes) on the GC% and rep%. These data were used for Figure 5 and Figures S4–S7.

Genome size data obtained from the Animal Genome Size Database [1] was utilized during the manual curation of the completeness of genomic data from NCBI and to the comparison of genome size and GC% between mammals and fish (Figure S1).

## 3. Results

Initially, we analysed the potential relationships between the chromosome size and its GC% across ray-finned fishes and compared them to one tunicate, one cephalochordate, all the three available lampreys, all the three available chondrichthyans, and to other vertebrates (amphibians, reptiles, birds, and mammals; Figure 1a, each point represents a single chromosome; graphs for each group are shown separately in Figure 1b–j). This shows that most fishes occupy the area with the lowest GC% and intermediate chromosome sizes (blue points). The single tunicate represented, the sea squirt (*Ciona intestinalis*, brown points in Figure 1b), occupies the actual lowest values of GC% and chromosome sizes and shows no association between these measures. The single cephalochordate represented, the Florida lancelet (*Branchiostoma floridae*, pink points in Figure 1c) is embedded approximately in the middle of the “fish area” surrounded by fish and shows a significant positive association between the GC% and chromosome size (better details in Figure 2a). In lampreys, the GC% and chromosome sizes are significantly positively associated (black points, Figure 1d, Figure 2b in better details). Chondrichthyans (black points, Figure 1e) but also sturgeon and gar, show a negative association between the GC% and chromosomes forming a curve resembling a hyperbole similarly to avian chromosomes (more details in Figure 3). In fishes (Figure 1a,f, blue), the linear approximation is mostly a suitable model representing their relationship between the chromosome size and GC%, however, all negative, positive, and no associations occur (more details in Figure 2 and below). Only eight of the nine currently available amphibian species are visualized here (Figure 1a,g, red) due to the extremely huge chromosome size in axolotl (*Ambystoma mexicanum*). All the amphibians are visualized in Figure 4 in proper details showing different levels of the negative association between the GC% and chromosome size not apparent at the resolution in Figure 1. Reptiles (Figure 1a,h, violet) with macro- and microchromosomes show a similar association of GC% with the chromosome size to chondrichthyans, basal fish lineages (sturgeon and gar), and birds. This means a negative relationship of GC% vs. chromosome size, behaving as if there was no association in large(r) (macro)chromosomes and a steep descent in GC% in microchromosomes. Birds (Figure 1a,i, orange) show a clear hyperbolic curve. Mammals (Figure 1a,j, green) show a negative relationship between GC% and the chromosome size with a varying strength similar to fishes. Violin plots of compositional differences between the selected fishes and mammals are in Figure S2.

### 3.1. Variability in Relationships between the Chromosome Size and GC% in Ray-Finned Fishes

Figure 2 shows representative species with diverse trends in their relationship between the chromosome size and GC%. The cephalochordate outgroup of all vertebrates, the lancelet (*B. floridae*), shows a positive correlation between GC% and the chromosome size that fits the linear model well (Figure 2a). Its haploid genome assembly size 513.461 Mb corresponds to the smallest teleosts and the size of its nineteen haploid chromosomes ranging between 17.12 and 35.34 Mb. The jawless outgroup of other vertebrates, the lamprey (*Lethenteron resissneri*) shows a negative correlation between GC% and the chromosomes size fitting the linear model well (Figure 2b). Its haploid genome assembly size is 1063.01 Mb and corresponds to the average teleosts, however, the size of its 72 haploid chromosomes ranges between 4.5 and 26 Mb. The remaining fish species depicted show diverse associations of diverse strength or no association at all. The non-teleost reedfish (*Erpetoichthys calabaricus*, Figure 2c) has a huge (haploid) genome of 3209 Mb separated into only eighteen chromosomes ranging in their size between 88.37 and 350.1 Mb (the largest chromosome is almost as large as the entire genome of pufferfishes, which have a reduced genome size). Its regression line is clearly decreasing, however, due to the extremely different scales of the X and Y axes, the line slope is close to zero. The relationship fits the linear model well (Figure 2c). Following are two teleosts belonging to the superorder Protacanthopterygii [40] but showing different trends and evolutionary history. Namely, in the Northern pike (*Esox Lucius*, Figure 2d), GC% decreases almost linearly with the increasing chromosome size, thus fitting the linear model well. This species has a typical teleost haploid genome size and chromosome number (940 Mb and 25 chromosomes ranging between 22.6 and 55.4 Mb). Whereas in the Atlantic salmon (*Salmo salar*, Figure 2e), that underwent the salmonid specific whole genome duplication points in the graph, form a cloud that does not fit the linear model and with no obvious simple relationship between the variables. This species has the haploid genome size 2966.89 Mb in 29 chromosomes of size between 40 and 160 Mb. The same situation exists in two other salmonids in NCBI assembled to the chromosome level (*Salmo*, *Oncorhynchus*, but not in *Thymallus* and *Salvelinus* that both show a slightly negative association between GC% and the chromosome size, not shown). The next species analysed is the model organism zebrafish (*Danio rerio*, Figure 2f) of the basal teleost lineage Ostariophysi [40], known to have a rather larger [1,41] and AT-enriched genome, e.g., [33]. Its haploid genome assembly of about 1408 Mb is divided into 25 chromosomes (2 n = 50) ranging in size between 37.5 and 59.58 Mb. Excluding the extreme, rightmost point of the graph the GC% oscillates around the mean independently of the chromosome size and the relationship does not fit a linear model. Other cypriniform fishes analysed (*Cyprinus* and *Carassius*, not shown) show similar trends, i.e., no association between the chromosome size and GC%. Similarly, in the Japanese rice fish (*Oryzias latipes*, Figure 2g), Beloniformes, the lower GC% appears to occur in larger chromosomes, but the relationship cannot be described in simple terms and does not fit the linear model well. So far, a unique situation among teleosts occurs in toadfish (*Thalassophryne amazonica*, Figure 2h) of the order Batrachoidiformes [40]. This species shows a significantly positive association between GC% and the chromosome size, similarly to the cephalochordate and a large haploid genome for a teleost, 2446.59 Mb with 23 large chromosomes ranging in size between 36.25 and 175.46 Mb. These traits together with its GC-richness (42.02%) resemble salmonids. Furthermore, the Southern platyfish (*Xiphophorus maculatus*, Figure 2i) of the order Cyprinodontiformes shows a tight linear relationship between GC% and the chromosome size (R^2^ = 0.7896) with points distributed visibly along the decreasing line. Finally, we selected pufferfish (*Takifugu rubripes*, Figure 2j) representing the order Tetraodontiformes with the smallest vertebrate genomes. It might appear that the shape of the line is influenced mainly by the two outer points. However, the decreasing tendency holds (although not that strong) without them and their negative association fits the linear model well.

### 3.2. Basal Fish Lineages Show a Similar Relationship between the Chromosome Size and GC% to Birds and Some Reptiles

In the two non-teleost ray-finned fishes and three chondrichthyans with available genomes assembled to the chromosome level (four shown here, Figure 3a–d), the GC% is inversely related to their chromosome size as in some reptiles (a snake and a turtle shown here, Figure 3e–f) and in birds (four shown here, Figure 3g–j). This relationship results in a hyperbolic curve in all the species analysed here. The curve is “smoother” for birds than in these basal fish lineages and the selected reptiles, where points representing single chromosomes are more scattered. In chondrichthyans, reptiles, and birds the chromosome size around 50 Mb represents a breakpoint behind the fact that the GC% becomes independent of the chromosome size. Sterlet and gar have this breakpoint around 20 Mb.

### 3.3. Genome Size Drives GC% in Amphibians but Inversely than Expected

Amphibians greatly differ in their genome size and salamanders are known to reach the second highest vertebrate genome sizes (116 and 118 Gb [42]). This fact highly influences the availability and also quality of amphibian genome assemblies in the NCBI database [43], whereas cytological data (C-values is pg) are very rich and provide robust evidence on the genome size in anurans and urodels [1]. The mean C-value of anurans and urodels is 3.59 pg and 35.21, respectively [1]. Our results show interesting consequences of this dynamic genome size evolution in amphibians for their GC%. Here, even such a low sample size (nine species) enabled to distinguish three distinct groups, partly phylogenetically independent, according to their GC%, chromosome size, and associations thereof (Figure 4):

1. A single salamander species with its giant and extremely GC-rich chromosomes (and genome) showing a weak negative association between GC% and the chromosome size (R^2^ = 0.32; genome size 32,396.4 Mb, GC ~ 46.5%).
2. Three caecilians and two frogs (pelobatid and bufonid) with an intermediate GC% and chromosome sizes (and intermediate genome sizes between 3779.43 and 5319.24 Mb, GC ~ 43%–44%) showing no association in two caecilians (R^2^ = 0.066 in *Rhinatrema*, R^2^ = 0.021 in *Geotrypetes*) to a significant negative association in the remaining caecilian *Microcaecilia* and two anurans (R^2^ = 0.63−0.72) between GC% and the chromosome size.
3. Three remaining frogs (two pipids and a pyxicephalid) with a significantly (R^2^ = 0.5837, 0.6689, and 0.7824) negative association between GC% and the chromosome size (the smallest genomes between 1451.3 and 2718.43 Mb, GC ~ 39%–40.5%).

This indicates that GC% increases together with the genome size and the genome size appears to be the determinant of this peculiar situation.

### 3.4. GC% vs. Repeats Proportion (%) and Chromosome Size

To explore any potential relationship between the chromosome size and their corresponding GC%, rep%, and GC% of repeats, we calculated these measures for each chromosome separately in 43 fish and fish-like species and in eleven mammals. These data were extracted from our tool EVANGELIST [34] and represent individual sliding windows along chromosomes and their GC% and rep% values. The results are pictured as scatter plots for each chromosome organized as tiles in Supplementary online data S4–S7 and on the GitHub online repository of our tool EVANGELIST (https://github.com/bioinfohk/evangelist_plots/tree/master/rep%25_vs_GC%25 (accessed on 31 January 2021)). These plots show several patterns in the relationships between GC% and repeats%, but no obvious association of GC%, rep%, and GC% of repeats with the chromosome size. The distribution of repeats percentage is asymmetric for each chromosome, while typically unchanging throughout the species and independent of the chromosome size. This can be seen from the position of the median of rep% (vertical line) in each tile. The same holds for the position of regression lines (similar in shape and position within the species). We also analysed the relationship between the repeats proportion at the level of entire chromosomes extracting the median rep% for each chromosome and it size (Figure 5). This is first shown in three cyprinids (goldfish, carp, and zebrafish, Figure 5a) differing in chromosome counts but similar genome sizes, where a significant (R^2^ = 0.46) negative association exists in goldfish, a very slight positive association in zebrafish, and no association at all in carp. Two compact tetraodontiform genomes show a moderate positive association in tetraodon and no association in fugu (Figure 5b). Another complex situation is shown in the Northern pike and three salmonids, where whole-genome duplication might have played an important role (Figure 5c). Interestingly, there is no clear association between the chromosome size and rep% even in microchromosomes (Figure 5d), where GC% is otherwise negatively associated with the chromosome size. Here, however, the availability of repeats libraries and hence, the quality of the repeat-masking process needs to be considered particularly in basal fish lineages (sturgeon and gar).

## 4. Discussion

Chromosome sizes greatly differ among currently sequenced chordates. In parallel to chromosome sizes, genome sizes and proportions of repeat also differ considerably. Despite some mostly superficial similarities, each major lineage of vertebrates occupies its own specific cytogenomic “niche” composed of a unique combination of cytogenomic traits.

### 4.1. Genome Size and Chromosome Numbers Do Not Entirely Explain the Difference in GC Evolution between Fish and Mammals

The meta-analysis of currently available data on genome size and GC% in vertebrates [1,43] shows that fish genomes are, despite their additional teleost-specific genome duplication [44], about two to three times smaller than mammalian genomes (Figure S1). However, both fish and mammals have converged to very similar diploid chromosome numbers (2 n) despite this difference in their genome evolution. Namely, the means and the medians of 2 n are 48–50 for fish [8] and 46–47 for mammals [1]. With these chromosome counts and fish genome size two to three times smaller than in mammals, this would mean roughly double the recombination rate per megabase (Mb) of DNA in fish in comparison with mammals [19]. This should result in higher GC contents in fish according to the concept of GC-biased gene conversion (gBGC) suggesting that the GC-richness of the mammalian genome is positively associated with higher recombination rates [18]. However, this is not the case—fish genomes are GC-poorer than the mammalian ones (Figure S1). Moreover, fish genomes are AT/GC homogenous in comparison with AT/GC heterogeneity in mammals as we show here in Supplementary, in our previous study in this special issue [34], as well as in [35]. This implies that the basic quantitative traits (genome size and 2 n) are not the only clues to the differences in nucleotide composition between fish and mammals and that another component must be involved. This component might be the repeatome, i.e., all repeated DNA sequences including also the simple tandem repeats, etc. [45] or specifically the mobilome, i.e., the sum of all mobile elements *sensu* [46]. The large-scale view of associations between the chromosome size vs. GC is similar in both mammals and fishes (Figure 1).

### 4.2. The Role of Chromosome Size Combines with the Influence of Repeats

The situation in chondrichthyans, sturgeon, and gar demonstrated here in comparison with birds indeed indicates an important role of the chromosome size on GC% of the entire genome. This is impacted by the presence of microchromosomes in all these species. The larger genome size in chondrichthyans and sturgeon can explain, in line with the concept of gBGC, their overall lower GC% (Figure 3). Despite this similar karyotype organization (numerous tiny microchromosomes and clearly larger macrochromosomes), the AT/GC heterogeneity of macrochromosomes has evolved only in gars [33] and not in sturgeons [47] and paddlefishes [48]. The closest living relatives of the AT/GC heterogeneous gars, the bowfin, possess the typical teleost-like karyotype in terms of AT/GC homogeneity and the absence of the microchromosomes [49]. On the other hand, both these ancient fish and birds show the independence of GC% from the chromosome size in their macrochromosomes (Figure 3), although the GC% of the mammalian chromosomes larger than 50–100 Mb is still negatively associated with the respective chromosome size (not shown here). This finding in both basal fish lineages and birds is contradictory to the concept of GC-biased gene conversion (gBGC) [18].

Salmonids and amphibians may provide another clue to understanding the relationship between the genome size and GC%. As shown here at the chromosomal level (Figure 5c), in another paper in this special issue [50] and earlier [30] at the genome level, there is no simple association between these two measures in salmonids. This can be at least partly explained by their extreme repeat content: 58%–60% in the Atlantic salmon [51], ~52% transposable elements in *Coregonus* sp. “Balchen” from the Lake Thun [52], 57.1% of the rainbow trout genome [53], RepeatMasker associated 53.07% of the genome with interspersed repeats, and masked 56.48% of the genome as repeat-derived in the Chinook salmon [54], etc. These high proportions of repeats belong to the highest ones among fishes and found in any vertebrate [51,55], similar to the only salamander species with a sequenced genome to data, the axolotl (*A. mexicanum*) with its 65.6% of repetitive sequences [56]. The impact of repeats on the GC% of the entire genome might be similar to that in zebrafish and some other cyprinids, where repeats occur in high proportions as well, 52.2% in Zv9 [41]. However, repeats in most cyprinids are GC-poor [30], whereas those in salmonids might be GC-rich, a possibility that needs to be explored. Both Class I and II transposons are almost equally represented in the sequenced salmonids. Therefore, it is currently impossible to ascribe the potential GC-enrichment to one of them as the dominant one *sensu* [30]. In axolotl, however, distinct long terminal repeat (LTR) retroelement classes and endogenous retroviruses made up the largest portion of the repetitive sequences and included elements of more than 10 kb in length [56]. This shows the broad repertoire in repeats composition in lineages with giant genomes and the need to explore them. The entire genomics of polyploid animals is still in its infancy and hence, important new facts can be expected soon [57] since new genomes have been sequenced in the meanwhile and others are on the way.

### 4.3. Nucleotide Composition Investigations in Fish

The currently most plausible findings link the genome GC content with the chromosome size and with life history traits, where the effective population size (Ne) is the most important indicator of the strength of the gBGC [18,20]. For fish, there is no systematic analysis of GC% based on purely genomic data as are available for mammals [20,21,22], birds [23,24], and reptiles [25,26]. One study assessing GC% based on non-genomic determination showed a slightly higher GC% in marine and migrating marine fish species [58]. Hence, we can merely indirectly assess the above mentioned factors with correlatives with GC% known for fish. Genome size is one of them and it is negatively correlated with the genomic GC% in fish except for salmonids [30]. Genome size was shown to negatively correlate also with the Ne and this association was demonstrated to have resulted in a larger genome in freshwater ray-finned fish [59]. This is in line with the aforementioned results on the effect of environment and lifestyle in fish [58]. However, the reduced Ne presumably resulting in larger and more complex genomes in freshwater fish [59] are contradictory to the situation in chondrichthyans, which are marine [40] and possess large genomes [43], but were not included in the study [59]. Another recent study indicated that the fish species distributed at higher latitude might have a small long-term Ne [60]. A link between Ne and the transposon evolution/transposition intensity was also indicated [61,62]. Moreover, there is a highly complex and ambiguous relationship between the transposons insertion and accumulation and the recombination rate (summarized in [63]).

Crucial results come from the compositional biology of mammalian transposons. There are indications that the base composition is evolving under selection and may be reflective of the long-term co-evolution between non-LTR retrotransposons and their host [64]. These authors also hypothesise that the coexistence of elements with drastically different base compositions suggests that these elements may be using different strategies to persist and multiply in the genome of their host. Similarly, other authors proposed that in addition to gBGC, there may be additional, still uncharacterized molecular mechanisms that either preserve genomic regions with biased nucleotide compositions from mutational degradation or fail to degrade such inhomogeneities in specific chromosomal regions [65].

## 5. Conclusions

To sum up, fishes show an immense diversity in their genome organisation and in the mutual relationships between their major genomic traits (GC% and genome size). On the other hand, their diploid chromosome numbers are relatively conserved [8], which means that their chromosome sizes need to decrease with the genome compaction observed in modern fish lineages. This resulted in an increased recombination rate as evidenced in the literature [19] and in a GC-enrichment, however, without AT/GC heterogeneity. Hence, modern fish lineages reached the smallest genome sizes among vertebrates despite their teleost-specific whole genome duplication [44]. Birds increased their absolute GC% and acquired or retained their AT/GC heterogeneity and substantially decreased their genome size, at least partly thanks to their microchromosomes (they increase the recombination rate that leads to genome compaction [66]). In contrast, basal fish lineages with microchromosomes do show a certain GC-enrichment, but they retained larger genome sizes despite the increased recombination rate mediated by microchromosomes that should have led to genome compaction similarly as in birds. Finally, mammals substantially expanded their genomes without any additional whole genome duplication and acquired or retained the AT/GC heterogeneity without any significant increase in their GC%. Therefore, mammals rather redistributed their genomes to GC-rich and GC-poor regions and accumulated them separately based on their GC%. The exact role and extent of GC-biased gene conversion in shaping the genome composition across vertebrates needs to be clarified and further investigated with the newly available resources. The quantitative approach to this issue, presented here, opens new possibilities to address related questions.

## Data Availability

https://github.com/bioinfohk/evangelist_plots (accessed on 31 January 2021).

## Supplementary Materials

The following are available online at https://www.mdpi.com/2073-4425/12/2/312/s1. Figure S1: Meta-analysis of GC% (A) and genome size (B) in fish (red) and mammals (green). Data from NCBI/genome and www.genomesize.com. Each point represents an entire genome; Figure S2: GC% of individual chromosomes in one lancelet (left, pink) and six ray-finned fishes (six blue violin plots) with diversified associations between the chromosome size and GC% compared with seven mammals (green violin plots). The overall comparison of GC% and genome size in fish and mammals can be found in Figure 1. The colour-code corresponds to Figure 1; Figure S3: GC% of individual chromosomes in lineages with microchromosomes—four ancient fish and in reptiles and birds. The left four violin plots are two chondrichthyans (thorny skate and bamboo shark in black), two basal ray-finned fishes (sterlet and spotted gar in blue), two violin plots in the middle are reptiles (rattlesnake and tortoise purple), and four right violin plots are birds (the Muscovy duck, chicken, the American flamingo, and zebra finch in yellow). The colour code corresponds to Figure 1; Figure S4: *Clupea harengus* GC% vs. repeats% in each chromosome. The chromosome size decreases from 33 to 12.4 Mb. Neither substantial changes in repeats% nor GC% of repeats occur with the decreasing chromosome size as predicted by the gBGC concept. A similar pattern was identified in all the three salmonids analysed here. The vertical line represents the median of rep%; Figure S5: *Danio rerio* GC% vs. repeats% in each chromosome. The chromosome size decreases from 78 to 37.5 Mb. Such a pattern has so far been identified only in this species. The vertical line represents the median of rep%; Figure S6: *Lepisosteus oculatus* GC% vs. repeats% in each chromosome. The chromosome size decreases from 73.2 to 5.4 Mb (we excluded the smallest chromosome with size 294 kb). Such a pattern occurs in numerous species; Figure S7: *Erpetoichthys calabaricus* GC% vs. repeats% in each chromosome. The chromosome size decreases from 350 to 88 Mb. The vertical line represents the median of rep%.
