# Supplementary material for: Quantitative Approach to Fish Cytogenetics in the Context of Vertebrate Genome Evolution
Source: Genes (Basel). 2021 Feb 22;12(2):312. doi: 10.3390/genes12020312 (PMC7926999; doi:10.3390/genes12020312)
Supplement: Supplementary file 1 [file genes-12-00312-s001.zip › genes-1030745-supplementary figures.docx]

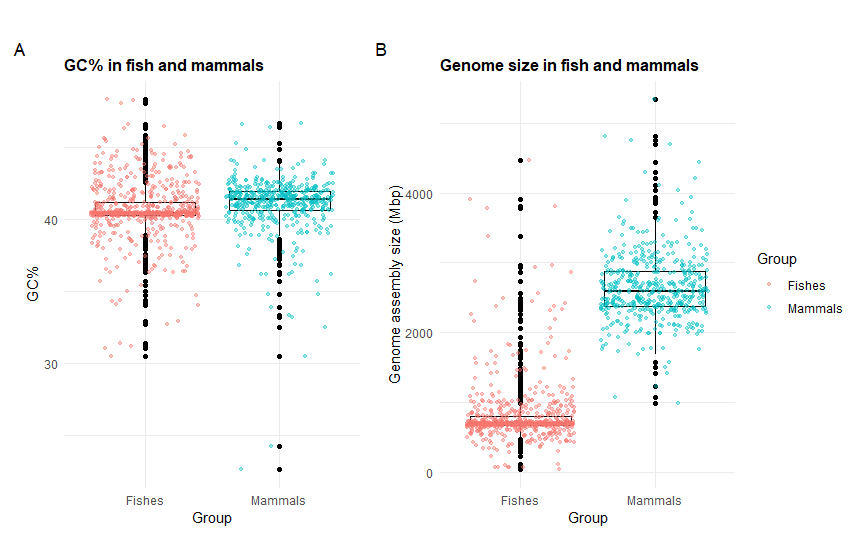


Fig. S1 Meta-analysis of GC% (A) and genome size (B) in fish (red) and mammals (green). Data from NCBI/genome and [www.genomesize.com](http://www.genomesize.com). Each point represents an entire genome


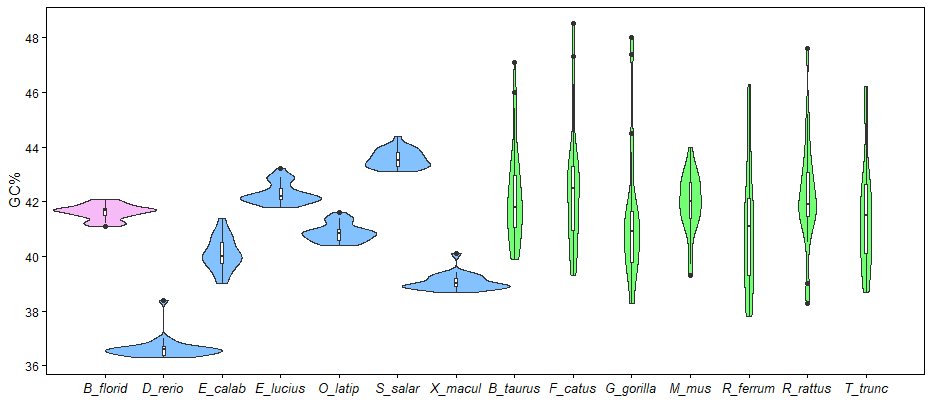


**Figure S2.** GC% of individual chromosomes in one lancelet (left, pink) and six ray-finned fishes (six blue violinplots) with diversified associations between chromosome size and GC% compared with seven mammals (green violinplots). The overall comparison of GC% and genome size in fish and mammals can be found in Fig. 1. The colour-code corresponds to Figure 1.


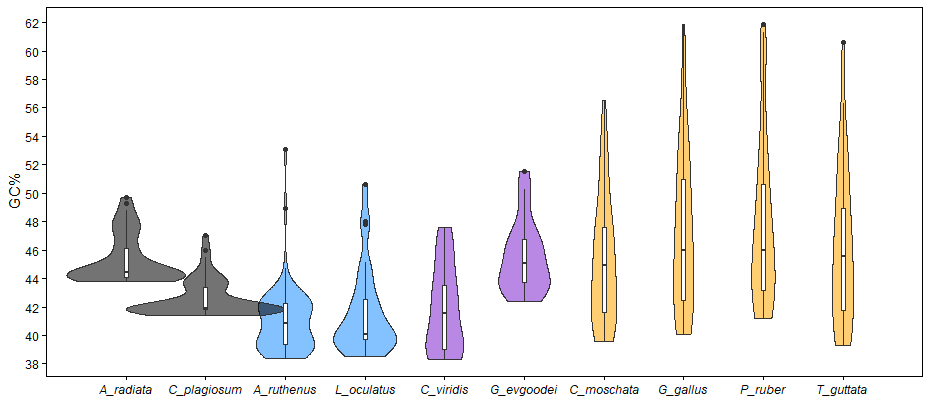


**Figure S3.** GC% of individual chromosomes in lineages with microchromosomes - four ancient fish and in reptiles and birds. Left four violin plots are two chondrichthyans (thorny skate and bamboo shark in black), two basal ray-finned fishes (sterlet and spotted gar in blue), two violin plots in the middle are reptiles (rattlesnake and tortoise purple) and four right violin plots are birds (the Muscovy duck, chicken, the American flamingo, and zebra finch in yellow). The colour code corresponds to Figure 1.


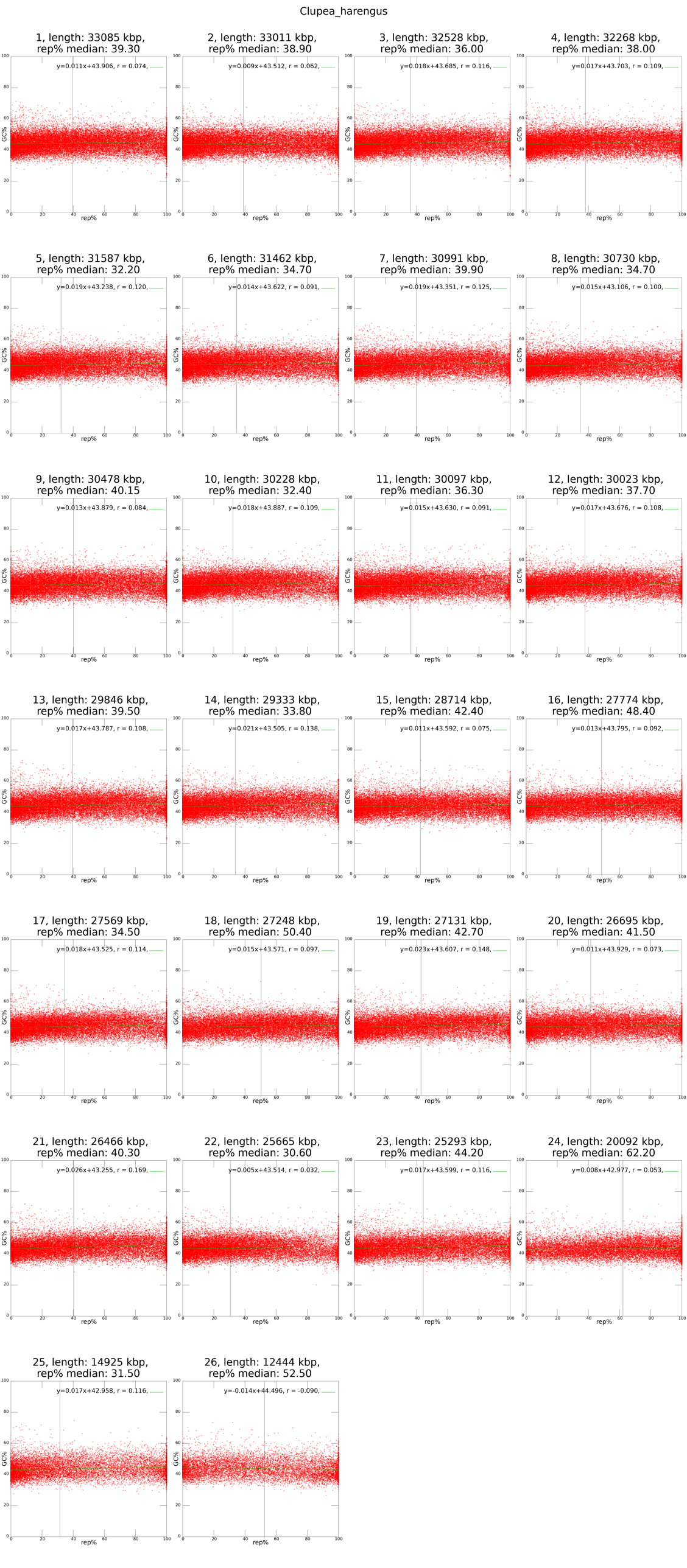


**Figure S4.** *Clupea harengus* GC% versus repeats% in each chromosome. Chromosome size decreases from 33 to 12.4 Mb. There occur neither substantial changes in repeats% nor GC% of repeats with the decreasing chromosome size as predicted by the gBGC concept. Similar pattern was identified in all three salmonids analysed here. The vertical line represents the median of rep%.


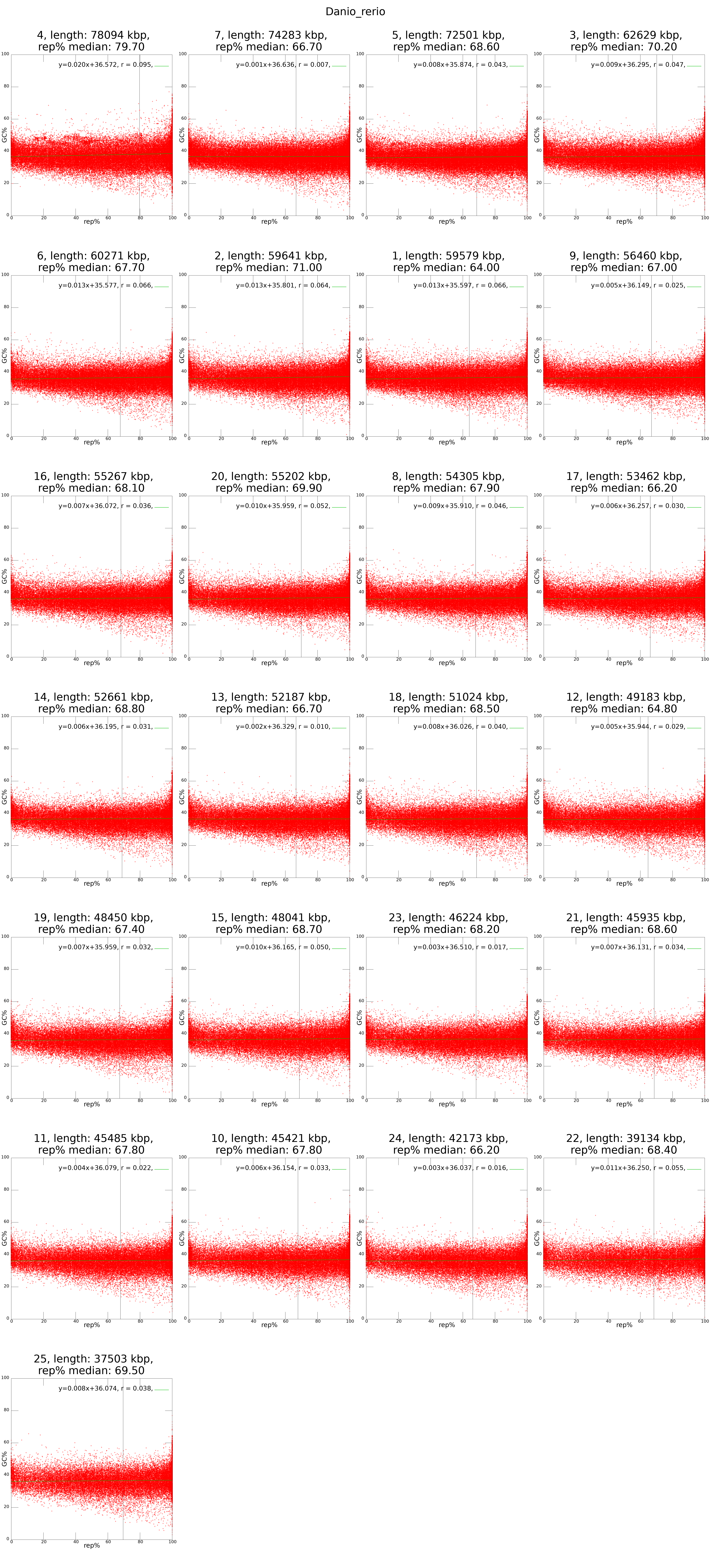


**Figure S5.** *Danio rerio* GC% versus repeats% in each chromosome. Chromosome size decreases from 78 to 37.5 Mb. Such a pattern has so far been identified only in this species. The vertical line represents the median of rep%.


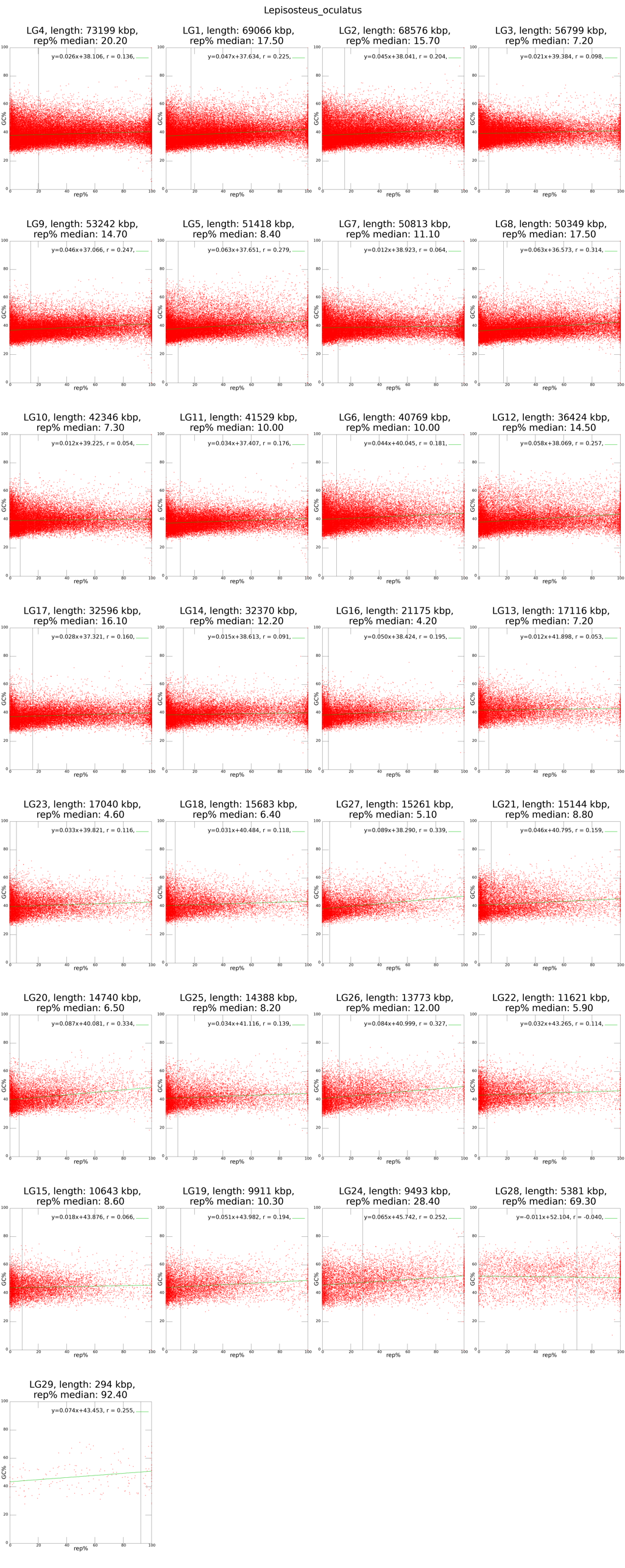


**Figure 6.** *Lepisosteus oculatus* GC% versus repeats% in each chromosome. Chromosome size decreases from 73.2 to 5.4 Mb (we excluded the smallest chromosome with size 294 kb). Such a pattern occurs in numerous species.


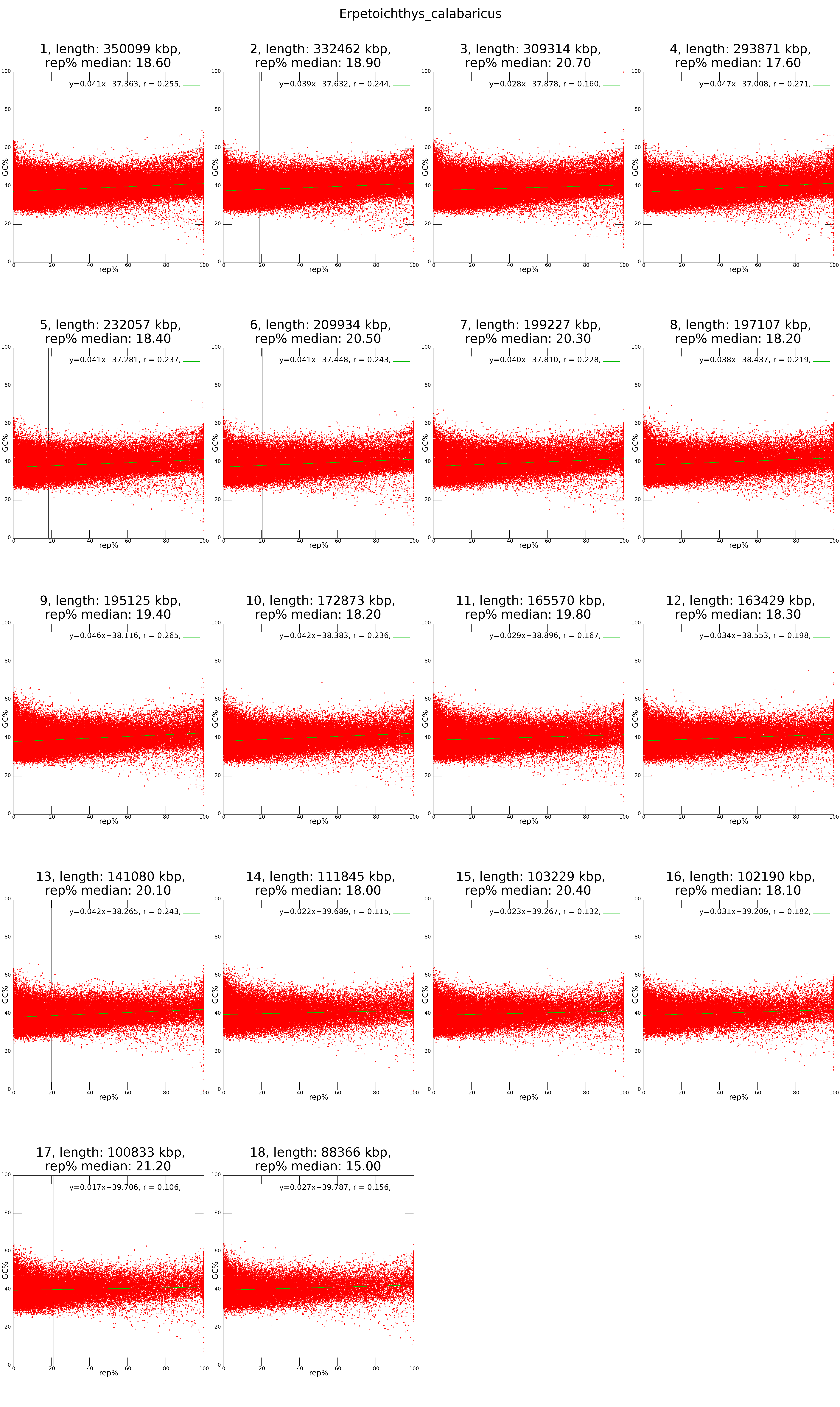


**Figure 7.** *Erpetoichthys calabaricus* GC% versus repeats% in each chromosome. Chromosome size decreases from 350 to 88 Mb. The vertical line represents the median of rep%.
